# Preoperative binocular vision characteristics in the age-related cataract population

**DOI:** 10.1186/s12886-022-02418-7

**Published:** 2022-04-27

**Authors:** Qing-Qing Tan, James S. Lewis, Chang-Jun Lan, Xuan Liao, Xiao-Li Tang, Jingyun Wang, Mitchell M. Scheiman

**Affiliations:** 1 Department of Ophthalmology, Affiliated Hospital of North Sichuan Medical College, 1 Mao Yuan South Road, Nanchong, Sichuan China; 2 Department of Ophthalmology and Optometry, North Sichuan Medical College, Nanchong, Sichuan China; 3 Graduate Programs in Biomedicine, Salus University, Elkins Park, PA USA; 4 Pennsylvania College of Optometry at Salus University, Elkins Park, PA USA; 5 State University of New York, College of Optometry, New York, NY USA

**Keywords:** Binocular vision anomaly, Convergence insufficiency, Age-related, Cataract

## Abstract

**Background:**

This study is the first part of the “Binocular Vision Anomalies after Cataract Surgery” study that aimed to investigate the impact of cataract surgery on binocular vision status in adults with age-related cataract. This study aimed to investigate the preoperative binocular vision status of participants with age-related cataract.

**Methods:**

Patients who elected to undergo bilateral cataract surgery (≥50 years of age) were recruited. Clinical measures of binocular vision including stereopsis, ocular alignment, fusional vergence, vergence facility, convergence amplitude and a symptom survey related to binocular vision anomalies were administered. A detailed classification protocol was established to identify the presence of binocular vision anomalies. The frequency of specific binocular vision anomalies and normative data of binocular vision measures were reported.

**Results:**

A total of 73 subjects were evaluated. No strabismus was detected in the cohort. Non-strabismic binocular vision anomalies were detected in 24 subjects (32.9%), of whom 18 (24.7%) had convergence insufficiency, 3 (4.1%) had basic exophoria, 2 (2.7%) had convergence excess, and 1 (1.4%) had fusional vergence dysfunction. Decreased vergence facility and convergence amplitude were more common compared to the pre-presbyopes (*P* < 0.01).

**Conclusion:**

Binocular vision problems, especially convergence insufficiency, are common in the adults with age-related cataract. The study results demonstrate that the lack of normative binocular vision data for the presbyopic population is a significant gap in the literature and suggest the need for a study of normative data for this population.

**Trial registration:**

The study was registered at ClinicalTrials.gov (NCT03592615, USA).

**Supplementary Information:**

The online version contains supplementary material available at 10.1186/s12886-022-02418-7.

## Background

Cataract surgery is the most commonly performed ophthalmic surgical procedure [1], and satisfactory monocular visual function is usually achieved in most patients [2]. However, studies indicate that post-surgical strabismic and non-strabismic binocular vision anomalies may occur leading to symptoms such as diplopia and asthenopia [3–8]. Diplopia and strabismus are the most commonly reported binocular vision-related complications after cataract surgery due to extraocular muscle paresis caused by infiltration anesthesia [3, 6]. In recent years, with almost universal application of topical anesthesia, the postoperative frequency of diplopia and strabismus is less common [9]. However, there were still indications in the literature that non-strabismic binocular vision anomalies may occur after topical anesthetic cataract surgery, including heterophoria [4, 5, 8], aniseikonia [4], and abnormalities in sensory and motor fusion [10]. Most studies have failed to provide data about pre-surgical binocular vision status, and/or have not performed a comprehensive binocular vision evaluation.

According to previous studies, the frequency of non-strabismic binocular vision anomalies varies from 13.2 to 56.2% in children or young adults [11–14]. This wide variability may be due to inconsistency in selection of diagnostic criteria [15], and the variability in binocularity among various age groups [16, 17]. As suggested by previous studies, age has an impact on binocular vision function, primarily because of the gradual loss of accommodation in presbyopia [16, 17]. However, major gaps in the current literature are a lack of data about the frequency of binocular vision anomalies in the presbyopic population, and limited information about expected findings in the older adult.

In the only relevant study of older adults, Leat et al. [18] retrospectively reviewed the medical records of an optometry clinic population aged 60 and older. They calculated the frequency of abnormal binocular vision test results including strabismus or phoria, incomitance, poor pursuits, and remote near point of convergence. They found that the frequency of any abnormal binocular vision test was 41 to 51% among different age subgroups. Leat et al.’s study [18] was the first reporting frequency data for abnormal binocular vision test results in older adults. While useful, the results unfortunately did not answer the question of what the frequency of binocular vision disorders is in older adults. Binocular vision disorders are considered syndromes, and therefore rather than a single test, the diagnosis is based on a series of test results [19]. As suggested by Scheiman and Wick, such tests include a minimum database consisting of the near point of convergence, the cover test at distance and near, and step vergence ranges at distance and near [19].

This study was designed to prospectively address one of the gaps in the literature by determining the frequency of binocular vision disorders in patients scheduled for bilateral cataract surgery, using well-defined clinical criteria, and a comprehensive test battery. In a follow-up paper we will compare the frequency of binocular vision anomalies and binocular vision measures before and after cataract surgery and determine risk factors that could predict those patients who might experience new binocular vision anomalies after cataract surgery. Herein, we present baseline data from a cohort of older presbyopes scheduled for bilateral cataract surgery.

## Methods

### Study design and subjects

This prospective study followed the tenets of the Declaration of Helsinki, and the study protocol was approved by the Institutional Review Board of Salus University (HQT1809). Patients with age-related cataract were consecutively recruited at The Eye Institute of Pennsylvania College of Optometry at Salus University and the medical practice of James *S. Lewis*, MD. (Elkins Park, PA). Written informed consents were obtained from all participants before any study testing was administered. The study was registered at ClinicalTrials.gov (NCT03592615, USA).

Major eligibility criteria included: ≥50-years-old, patient has elected to undergo bilateral cataract extraction and intraocular lens implantation, best corrected visual acuity ≥20/80 in each eye. Patients with any ocular pathology other than cataract or strabismus, or a history of previous ocular surgery related to the extraocular muscles, intraocular lens implantation or laser refractive surgery were excluded.

A comprehensive battery of binocular vision tests (Table 1) was administered to all the subjects before cataract surgery. A detailed diagnostic classification protocol (Table 2) was used to identify and classify the presence of a binocular vision anomaly.

**Table 1:** Descriptions of outcome measures

Binocular function assessed	Equipment/units of measure	Administration details
**Stereoacuity**	Randot Stereo Test/ arc seconds (“)	Begin with global stereotest, then contour test was performed to determine the exact stereoacuity that could be measured up to 20″.
**Ocular deviation**	Occluder, prism bar/Δ, exodeviations recorded with a minus sign, esodeviations with plus sign.	Unilateral cover test followed by the alternate prism cover test. Near and distance ocular deviations were measured, respectively.
**Fusional vergence**	Horizontal prism bar/Δ.	A target (thin vertical line) was held 40 cm away and a hand-held prism bar was used. Near and distance, positive fusional vergence (BO) and negative fusional vergence (BI) were measured by slowing increasing the amount of prism. The result was 4 measures: distance BI, distance BO, near BI, near BO. Each measure was recorded as 3 values: blur/break/recovery.
**Vergence facility**	12 BO/3 BI prism flipper/CPM.	A target (thin vertical line) was held 40 cm away and a hand-held prism bar was used, and the prism side with most difficulty was recorded. Near and distance vergence facility were measured, respectively.
**Near point of convergence**	Near Point Rule with narrow vertical line**/**cm.	Near Point Rule held against brow, target slowly moved towards eye to until first sustained report of double vision (break), then moved away until recovery of single vision (recovery). Results were recorded as 2 values: near point of convergence break and recovery.
**Convergence Insufficiency Symptom Survey (CISS)**	15-item questionnaire/score from 0 up to 60.	Patient was asked to complete the survey. A higher score indicating more symptoms.

Δ = prism diopter, *BO* Base-out, *BI* Base-in, *cm* Centimeters, *CPM* Cycles per minute

**Table 2:** Diagnostic criteria for non-strabismic binocular vision anomalies (adapted from Scheiman and Wick [19])

**Convergence insufficiency**	
Symptoms:	
Associated with reading or other near tasks and generally worse at end of day. The most common symptoms include asthenopia and headaches, intermittent diplopia.	
Signs:	
1) Exophoria at near (≥ 4 ∆), greater than distance.	
2) Receded near point of convergence break with accommodative target ≥6 cm.	
3) Insufficient positive fusional vergence at near (i.e., failing Sheard’s criterion or positive fusional vergence ≤15∆ base-out break).	
4) Vergence facility ≤9 cycle per minute (CPM) (difficulty with 12 ∆ Base out prism).	
For diagnosis: 2-sign criterion: sign 1) plus sign 2) or 3); 3-sign criterion: all the first three criteria are mandatary.	
**Divergence insufficiency**	
Symptoms:	
Associated with distance viewing. The most common symptoms include intermittent diplopia for distance, headache, and eyestrain.	
Signs:	
1) Esophoria greater at distance than near by ≥3 ∆.	
2) Reduced negative fusional vergence break ≤6 ∆ for distance.	
3) Vergence facility ≤9 cycle per minute (CPM) (difficulty with 3 ∆ Base in prism).	
For diagnosis: Criterion 1 is mandatory with a minimum of one criterion from 2 and 3.	
**Convergence excess**	
Symptoms:	
Associated with reading or other near tasks and generally worse at end of day. The most common symptoms include asthenopia, headaches and intermittent diplopia.	
Signs:	
1) Esophoria greater at near than distance by ≥3PD.	
2) Reduced negative fusional vergence break ≤8 ∆ at near, or if Sheard’s criterion is not met (that the negative fusional vergence measures less than twice the magnitude of the near phoria).	
3) vergence facility ≤9 CPM (difficulty with 3 ∆ Base in prism).	
For diagnosis: Criterion 1 is mandatory with a minimum of one criterion from 2 and 3.	
**Divergence excess**	
Symptoms:	
Associated with distance viewing than near. The most common complaint is related to cosmesis.	
Signs:	
1) High exophoria or intermittent exotropia at distance with the magnitude of the deviation for distance greater than near of ≥10 ∆.	
2) The proportion of time the deviation is intermittent is greater at distance than at near on the office control score.	
3) Low negative fusional vergence break ≤8 ∆ for distance.	
For diagnosis: Criterion 1 is mandatory.	
**Fusional vergence dysfunction**	
Symptoms:	
Associated with reading or other near tasks and generally worse at end of day. The most common symptoms include asthenopia and headaches, blurred vision and difficulty concentrating on near visual tasks.	
Signs:	
1) Reduced negative fusional vergence ≤8 ∆ and positive fusional vergence ≤15 ∆ break at near or if Sheard’s criterion is not met.	
2) vergence facility ≤9 CPM (difficulty with 3 ∆ base in and 12 ∆ base out prism).	
For diagnosis: All criteria are mandatory.	
**Basic esophoria**	
Symptoms:	
Associated with reading or other near tasks and with distant activities. The most common near point complaints include eyestrain, headaches, and blurred vision. Common symptoms associated with distance include blurred vision and diplopia, when watching television and in classroom.	
Signs:	
1) Equal magnitude of esophoria at distance and near (within 5 ∆ are considered equal).	
2) Reduced negative fusional vergence break ≤3 ∆ at distance and ≤ 8 ∆ at near.	
3) vergence facility ≤9 CPM (difficulty with 3 ∆ Base in prism).	
For diagnosis: Criteria 1 is mandatory with one out of the next two criteria.	
**Basic exophoria**	
Symptoms:	
Associated with reading or other near tasks and with near and distant activities. The most common near point complaints include eyestrain, headaches, and blurred vision. Signs:	
1) Equal amount of exophoria at distance and near (within 5 ∆ are considered equal).	
2) Receded near point of convergence break ≥6 cm with accommodative target.	
3) Reduced positive fusional vergence break ≤10 ∆ for distance and ≤ 15 ∆ at near. or if Sheard’s criterion is not met.	
4) vergence facility ≤9 CPM (difficulty with 12 ∆ Base out prism).	
For diagnosis: Criterion 1 is mandatory with two out of the next three criteria.	

### Outcome measurements

The eligibility assessment was performed using routine eye examination procedures to rule out ocular pathology other than cataract, refractive error, and strabismus. As described in Table 1, a comprehensive battery of binocular vision tests including stereopsis, ocular alignment, fusional vergence, vergence facility, near point of convergence, and a symptom survey for binocular vision anomalies was administered to all the subjects. All examinations were performed using the habitual prescriptions or trial frames that provided best corrected visual acuity. The testing protocol required about 25 min. To evaluate the near point of convergence, a thin vertical line target mounted on a Near Point Rule was used. In the only previous report on the near point of convergence measurement for older presbyopes the procedure and target used was not described [18]. The standard target used in children or pre-presbyopes is a vertical column of 20/30 letters, but in pilot testing the presbyopic participants in this study were unable to see the line clearly. Thus, a thin vertical line was used based on a previous study [20] that compared the use of a vertical line target mounted on a Royal Air Force rule with N5 letter target, in which there was no significant difference in the result with the two targets. All participants were presbyopic in this study, so accommodative testing was not performed.

### Diagnostic criteria for classification of binocular vision anomalies

Diagnostic criteria were adapted from Scheiman and Wick [19] (Table 2). The only modification was to grade convergence insufficiency with a 3-sign criterion as recommended in previous studies, in which convergence insufficiency was sub-classified into 3-sign (definite), 2-sign (high suspect) and 1-sign (low suspect) [21]. The three clinical signs and their critical cut-offs used to grade convergence insufficiency include: exophoria at near ≥4 ∆, greater than distance; receded near point of convergence break ≥6 cm; and, insufficient positive fusional vergence at near (i.e., failing Sheard’s criterion or positive fusional vergence ≤15∆ base-out break). Classification criteria were defined as follows: 3-sign convergence insufficiency requires all the three clinical signs; 2-sign convergence insufficiency requires sign 1 plus sign 2 or 3; 1-sign convergence insufficiency requires only sign 1 [21]. In this study, the 2-sign and 3-sign criteria determined frequency data were reported. The unilateral cover test was used to determine if a constant or intermittent strabismus was present.

### Statistical analysis

A sample size calculation based on a McNemar’s test to compare the prevalence of non-strabismic binocular vision anomalies pre- and post-cataract surgery was performed using the Power and Sample Size Program (PS version 3.1.2). The prevalence of non-strabismic binocular vision anomalies in the adult population is 10.86% as reported by previous literature [13]. We proposed that a 15% change in prevalence is clinically meaningful based on expert opinion. The sample size calculation suggested that a total of 38 subjects would yield a power of 80% with a significance level of 0.05.

All analyses were performed using SPSS Statistics 25.0 with an alpha level of 0.05 to determine the statistical significance. A calculation for the frequency of binocular vision anomalies was performed. Continuous data were expressed by means and standard deviations (SD), while categorical data were expressed by percent or numeric. Before data analyses, a Shapiro–Wilk test was performed for all continuous variables to determine the data distribution. According to the data distribution, a Student’s *t*-test or Mann-Whitney U test was used to compare the clinical measures between participants with normal binocular vision and those with binocular vision anomalies. A one-sample *t* test was used to compare the obtained binocular vision measures to that reported elsewhere for pre-presbyopes. To specifically illustrate the Convergence Insufficiency Symptom Survey (CISS) scores for the presbyopes, a clustered bar chart was built based on the frequency that participants reported “fairly often” or “always” on each CISS item. In the chart, the 15 CISS symptoms were divided into performance-related and eye-related symptoms according to Barnhardt et al. [22].

## Results

### Participant characteristics

From January to November 2019, 74 participants met the eligibility criteria, of whom 1 participant failed to complete the outcome evaluation, thus 73 participants were included. The mean age was 70.2 years. Demographics and participant characteristics are shown in Table 3.

**Table 3:** Demographics and characteristics of the participants

Demographics and characteristics	Values
	Number
Gender (female/male)	57/16
Race (African American/Caucasian/Hispanic or Latino)	68/4/1
	**Mean ± SD**
Age (year)	70.2 **±** 6.7
Corrected distance visual acuity OD (LogMAR)	0.15 **±** 0.15
Corrected distance visual acuity OS (LogMAR)	0.16 **±** 0.15
Spherical equivalent OD (diopter)	−0.06 **±** 2.24
Spherical equivalent OS (diopter)	− 0.03 **±** 1.86
Stereoacuity (arc second)	160.68 **±** 160.17

*OD* Right eyes, *OS* Left eyes, *LogMAR* Logarithm of the minimum angle of resolution, *SD* Standard deviation

### Frequency of binocular vision anomalies

As shown in Table 4, of the 73 participants enrolled, no strabismus was detected, while 18 (24.7%) had 3-sign convergence insufficiency and 40 (54.8%) had 2-sign convergence insufficiency. There were a small percentage of participants with other binocular vision conditions such as convergence excess, basic exophoria, and fusional vergence dysfunction.

**Table 4:** Frequency of binocular vision anomalies in the study cohort

Condition	Frequency
Normal binocular vision	67.1% (49/73)
3-sign convergence insufficiency	24.7% (18/73)
2-sign convergence insufficiency	54.8% (40/73)
Basic exophoria	4.1% (3/73)
Convergence excess	2.7% (2/73)
Fusional vergence dysfunction	1.4% (1/73)

### Binocular vision data from participants with normal binocular vision

**Fig. 1:**
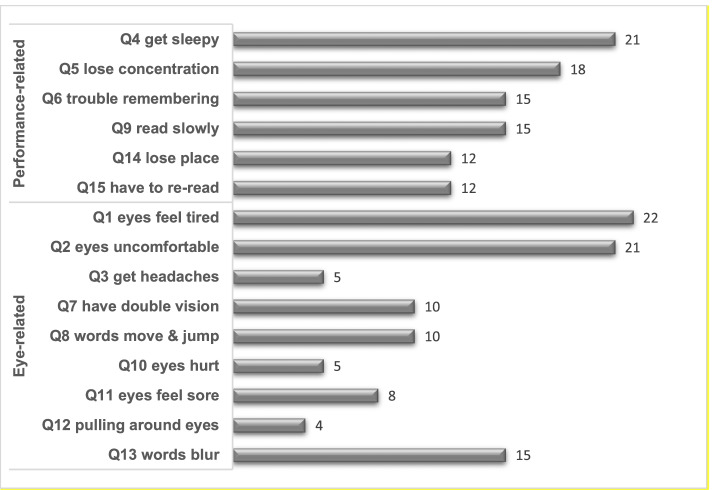
Percentage responding “fairly often” or “always”

In Table 5, the binocular vision data of the participants with normal binocular vision and those with non-strabismic binocular vision anomalies are compared. Significant differences were detected in near ocular deviation (*P* = 0.02), distance and near positive fusional vergence (*P* < 0.01), near vergence facility (*P* < 0.01) and the near point of convergence (*P* < 0.05), while no differences were detected in other binocular vision measures (*P* > 0.05). There was no significant difference in the mean CISS score between participants with normal binocular vison and those with non-strabismic binocular vision anomalies (20.0 vs. 17.7, *P* = 0.41). When CISS items were analyzed individually, performance-related and eye-related symptoms appeared to be equally reported by the 73 participants (Fig. 1).

**Table 5:** Binocular vision measures of the participants

Clinical measures	NBV (Mean ± SD) (*n* = 49)	NSBVA (Mean ± SD) (*n* = 24)	***P*** values
Age (year)	69.4 ± 6.5	71.9 **±** 7.0	0.13^§^
Corrected distance visual acuity OD (LogMAR)	0.15 ± 0.15	0.16 **±** 0.14	0.97
Corrected distance visual acuity OS (LogMAR)	0.14 ± 0.13	0.18 ± 0.17	0.41
Spherical equivalent OD (diopter)	−0.08 ± 2.28	0.01 ± 2.21	0.87^§^
Spherical equivalent OS (diopter)	− 0.16 ± 1.85	0.23 ± 1.87	0.40^§^
Stereoacuity (arc second)	172.1 **±** 165.6	137.3 **±** 149.0	0.32
Ocular deviation at distance (Δ)	− 0.9 ± 2.2	−1.2 ± 2.6	0.35
Ocular deviation at near (Δ)	−3.9 **±** 3.8	− 5.2 ± 7.7	0.02*
BI break at distance (Δ)	10.5 **±** 7.2	9.0 ± 6.6	0.22
BI recovery at distance (Δ)	7.2 **±** 5.8	5.2 ± 3.6	0.10
BO break at distance (Δ)	20.4 **±** 11.2	13.1 ± 9.2	< 0.01*
BO recovery at distance (Δ)	15.4 **±** 9.5	10.0 ± 8.9	< 0.01*
BI break at near (Δ)	14.0 **±** 5.7	11.4 ± 4.7	0.07
BI recovery at near (Δ)	10.3 **±** 4.0	8.7 ± 4.6	0.21
BO break at near (Δ)	30.6 **±** 12.6	11.8 ± 3.6	< 0.01*
BO recovery at near (Δ)	23.5 **±** 11.6	8.4 ± 4.7	< 0.01*
Vergence facility at distance (CPM)	6.8 ± 5.8	4.2 ± 4.5	0.06
Vergence facility at near (CPM)	11.5 ± 5.3	6.4 ± 4.8	< 0.01^§^*
Near point of convergence break (cm)	8.6 **±** 3.7	10.6 ± 3.6	0.02*
Near point of convergence recovery (cm)	10.6 **±** 4.4	13.1 ± 4.7	0.01*
CISS score	20.0 **±** 11.1	17.7 ± 11.1	0.41^§^

*NBV *Normal binocular vision, *NSBVA* Non-strabismic binocular vision anomalies, *OD* Right eyes, *OS* Left eyes, *LogMAR* Logarithm of the minimum angle of resolution; Δ = prism diopter, *BO* Base-out, *BI* Base-in, *cm* Centimeters, *CPM* Cycles per minute, *SD* Standard deviation, *CISS* Convergence Insufficiency Symptom Survey; *: the difference is statistically significant; §: a Student’s *t*-test was used for the marked comparison, while a Mann-Whitney *U* test was used for the rest of comparisons

## Discussion

Although the participants in this study were all diagnosed with cataracts requiring surgical intervention, the baseline data still provide valuable information for eye care clinicians regarding the presbyopic population with cataract. Among the 73 participants, no strabismus was detected, while non-strabismic binocular vision anomalies were common (32.9%), and the majority had convergence insufficiency (24.7%) based on a 3-sign diagnostic criterion.

The frequency of non-strabismic binocular vision anomalies reported in previous studies on pediatric or pre-presbyopic population varies from 13.2 to 40%, with convergence insufficiency the most frequently occurring disorder with a frequency ranging from 2.3 to 33% [11–14]. The wide variance among studies can be attributed to the age ranges of the studied populations, and inconsistency in the diagnostic testing and criteria used by investigators.

Comparison of these results to the existing literature was challenging for two reasons. First, there are limited normative data available for the near point of convergence, positive fusional vergence, and vergence facility in the literature for older presbyopic patients. We were unable to find any prospective study with these data, and only one retrospective study by Leat et al. [18] However, the study by Leat et al. did not include assessment of fusional vergence and vergence facility, and they did not report the actual frequency of binocular vision disorders. Rather, they reported the frequency of abnormal, individual test results for the cover test and near point of convergence. Hence, we were unable to directly compare our results to Leat et al.’s.

The second problem is that the previous studies of binocular vision in presbyopia did not include a comprehensive binocular vision evaluation. None of the previous studies evaluated all three necessary components of a binocular vision evaluation: eye alignment, near point of convergence and fusional vergence. For the few measures (ocular alignment at far and near, fusional vergence at far, vergence facility at near, convergence amplitude) that are available from previous studies [17, 23–25], there appears to be reasonably good agreement with our data.

However, when comparing our data to the expected finding in pre-presbyopes [26, 27], it is noteworthy that vergence facility was significantly slower in the presbyopic population. The expected values for near and distance recommended by Gall et al. for the young adults [26, 27] are 15 cycles per minute for near and 12 cycles per minute for distance respectively, while in the present study we found 11.5 cycles per minute for near (one-sample t test, *t* = − 4.63, *P* < 0.01) and 6.8 cycles per minute for distance (one-sample *t* test, *t* = − 6.32, *P* < 0.01). Previous studies revealed a lack of correlation between vergence facility and the disparity vergence range [28]. This is supported by the results in this study showing normal, mean fusional vergence ranges, yet a decreased mean vergence facility measure detected. These findings suggest that to adequately assess fusional vergence a clinician should measure both the amplitude (fusional vergence ranges) and vergence facility. The mean break for the near point of convergence in the present study was 8.6 cm compared to 2.5 cm suggested by Scheiman & Wick [19] for young adults (one-sample *t* test, *t* = 11.56, *P* < 0.01). Previous studies suggest that while there is an age-related decline in accommodative amplitude the convergence response remains relatively constant during the development of presbyopia [16, 17].

The CISS is a well-validated symptom survey designed to be used as an outcome measure before and after intervention for patients with convergence insufficiency [29–31]. In young adults, a score of ≥21 is considered significant, suggesting that the patient is symptomatic [30]. In this study, a mean CISS score of 20 was present and 42.9% participants showed CISS score > 21. However, it is important to remember that the CISS has been only validated for use in children and young adults. It has not been validated for use in presbyopic patients. In addition, in this study, there was no significant difference in the CISS score between participants with normal binocular vision and those binocular vision anomalies, and the performance-related and eye-related symptoms appeared to be equally reported as shown in Fig. 1. Thus, we speculate that the changes in the CISS of presbyopes may not only be related to changes in binocular vision, but also to ocular comfort and function. We suggest that the use of CISS for assessment of symptoms in the older presbyopic population should be used with caution until it is validated for this population.

Our data suggest, that if the current normative data are applied to the presbyopic population with cataracts requiring surgery, there is a high frequency of non-strabismic binocular vision anomalies. However, these data also suggest that new research should be directed at developing normative data for this population. There is also a significant need for prospective studies of the presbyopic population with no cataract or other eye disease.

There are limitations in this study: 1) Because our data were derived from the “Binocular Vision Anomalies after Cataract Surgery” study, in which the participants were all cataract patients, the results may not be extrapolated to the older adult without cataracts. However, given the high frequency of cataracts in older adults, these study data do provide a meaningful picture of binocular vision in older adults; 2) The data of grade and asymmetry in cataracts were not collected in this study, however, we had a rigorous visual acuity criterion during the recruitment, and our results suggested that there was no difference in visual acuity between the right and left eyes. Thus, we do not think this factor would bias our conclusions; 3) The majority of this cohort was African American, which makes generalization to other populations potentially problematic.

## Conclusions

The results from this study using both a comprehensive binocular vision assessment, and universally applied diagnostic criteria demonstrate that several clinical measures of binocular vision in the presbyopic population are significantly different from the young adult population, and the frequency of non-strabismic binocular vision anomalies particularly convergence insufficiency is common before cataract surgery. This finding suggests the need for future research to establish normative data for the presbyopic population and the frequency of non-strabismic binocular vision anomalies for this population.

## Supplementary Information

**Additional file 1.**

## Data Availability

All data generated or analysed during this study are included in this published article and its supplementary information files.

## Abbreviations

BAND: Binocular Vision Anomalies and Normative Data
CISS: Convergence Insufficiency Symptom Survey
BO: Base-out
BI: Base-in
CPM: Cycles per minute
LogMAR: Logarithm of the minimum angle of resolution
SD: Standard deviation

## References

[1] Wang W, Yan W, Fotis K, Prasad NM, Lansingh VC, Taylor HR (2016). Cataract surgical rate and socioeconomics: a global study. Invest Ophthalmol Vis Sci.

[2] Zhang JY, Feng YF, Cai JQ (2013). Phacoemulsification versus manual small-incision cataract surgery for age-related cataract: Meta-analysis of randomized controlled trials. Clin Exp Ophthalmol.

[3] Gawecki M, Grzybowski A (2016). Diplopia as the complication of cataract surgery. J Ophthalmol.

[4] Rutstein RP, Fullard RJ, Wilson JA, Gordon A (2015). Aniseikonia induced by cataract surgery and its effect on binocular vision. Optom Vis Sci.

[5] Chung SA, Kim CY, Chang JH, Hong S, Kang SY, Seong GJ (2009). Change in ocular alignment after topical anesthetic cataract surgery. Graefes Arch Clin Exp Ophthalmol.

[6] Nayak H, Kersey JP, Oystreck DT, Cline RA, Lyons CJ (2008). Diplopia following cataract surgery: a review of 150 patients. Eye (Lond).

[7] Dujic MP, Misailovic KR, Kovacevic MM (2005). Persistent strabismus after cataract extraction. Vojnosanit Pregl.

[8] Golnik KC, West CE, Kaye E, Corcoran KT, Cionni RJ (2000). Incidence of ocular misalignment and diplopia after uneventful cataract surgery. J Cataract Refract Surg.

[9] Yanguela J, Gomez-Arnau JI, Martin-Rodrigo JC, Andueza A, Gili P, Paredes B (2004). Diplopia after cataract surgery: comparative results after topical or regional injection anesthesia. Ophthalmology.

[10] Loba P, Rajska K, Simiera J, Wilczynski M, Omulecki W, Broniarczyk-Loba A (2015). The influence of a prolonged Interoperative period on binocular vision after bilateral cataract extractions. Eur J Ophthalmol.

[11] Ma MM, Yeo ACH, Scheiman M, Chen X (2019). Vergence and accommodative dysfunctions in Emmetropic and myopic Chinese young adults. J Ophthalmol.

[12] Hussaindeen JR, Rakshit A, Singh NK, George R, Swaminathan M, Kapur S (2017). Prevalence of non-Strabismic anomalies of binocular vision in Tamil Nadu: report 2 of Band study. Clin Exp Optom.

[13] Garcia-Munoz A, Carbonell-Bonete S, Canto-Cerdan M, Cacho-Martinez P (2016). Accommodative and binocular dysfunctions: prevalence in a randomised sample of university students. Clin Exp Optom..

[14] Scheiman M, Gallaway M, Coulter R (1996). Prevalence of vision and ocular disease conditions in a clinical pediatric population. J Am Optom Assoc.

[15] Cacho-Martinez P, Garcia-Munoz A, Ruiz-Cantero MT (2014). Is there any evidence for the validity of diagnostic criteria used for accommodative and Nonstrabismic binocular dysfunctions?. J Optom.

[16] Heron G, Charman WN, Schor CM (2001). Age changes in the interactions between the accommodation and Vergence systems. Optom Vis Sci.

[17] Palomo Alvarez C, Puell MC, Sanchez-Ramos C, Villena C (2006). Normal values of distance Heterophoria and fusional Vergence ranges and effects of age. Graefes Arch Clin Exp Ophthalmol.

[18] Leat SJ, Chan LL, Maharaj PD, Hrynchak PK, Mittelstaedt A, Machan CM (2013). Binocular vision and eye movement disorders in older adults. Invest Ophthalmol Vis Sci.

[19] Scheiman M, Wick B (2019). Clinical Management of Binocular Vision: Heterophoric, accommodative and eye movement disorders.

[20] Adler PM, Cregg M, Viollier AJ, Margaret WJ (2007). Influence of target type and Raf rule on the measurement of near point of convergence. Ophthalmic Physiol Opt.

[21] Ma MM, Long W, She Z, Li W, Chen X, Xie L (2019). Convergence insufficiency in Chinese high school students. Clin Exp Optom..

[22] Barnhardt C, Cotter SA, Mitchell GL, Scheiman M, Kulp MT (2012). Symptoms in children with convergence insufficiency: before and after treatment. Optom Vis Sci.

[23] Freier BE, Pickwell LD (1983). Physiological Exophoria. Ophthalmic Physiol Opt..

[24] Pellizzer S, Siderov J (1998). Assessment of Vergence Facility in a Sample of older adults with presbyopia. Optom Vis Sci.

[25] Ostadimoghaddam H, Hashemi H, Nabovati P, Yekta A, Khabazkhoob M (2017). The distribution of near point of convergence and its association with age, gender and refractive error: a population-based study. Clin Exp Optom..

[26] Gall R, Wick B, Bedell H (1998). Vergence facility: establishing clinical utility. Optom Vis Sci.

[27] Gall R, Wick B, Bedell HE, Pease PL (1995). Vergence facility: establishment of clinical norms. Opt Vis Sci.

[28] McDaniel C, Fogt N (2010). Vergence adaptation in clinical Vergence testing. Optometry (St Louis, Mo).

[29] Borsting EJ, Rouse MW, Mitchell GL, Scheiman M, Cotter SA, Cooper J (2003). Validity and reliability of the revised convergence insufficiency symptom survey in children aged 9 to 18 years. Optom Vis Sci.

[30] Rouse MW, Borsting EJ, Mitchell GL, Scheiman M, Cotter SA, Cooper J (2004). Validity and reliability of the revised convergence insufficiency symptom survey in adults. Ophthalmic Physiol Opt.

[31] Rouse M, Borsting E, Mitchell GL, Cotter SA, Kulp M, Scheiman M (2009). Validity of the convergence insufficiency symptom survey: a confirmatory study. Optom Vis Sci.

